# Contribution of Local Analysis Techniques for the Characterization of Iron and Alloying Elements in Nitrides: Consequences on the Precipitation Process in Fe–Si and Fe–Cr Nitrided Alloys

**DOI:** 10.3390/ma11081409

**Published:** 2018-08-11

**Authors:** Hugo P. Van Landeghem, Raphaële Danoix, Mohamed Gouné, Sylvie Bordère, Andrius Martinavičius, Peter Jessner, Thierry Epicier, Béatrice Hannoyer, Frédéric Danoix, Abdelkrim Redjaïmia

**Affiliations:** 1 Institut Jean Lamour, UMR 7198, CNRS, Université de Lorraine, Campus ARTEM, 2 allée André Guinier, F-54011 Nancy CEDEX, France; hugo.van-landeghem@grenoble-inp.fr (H.P.V.L.); andriusmart@gmail.com (A.M.); 2 LABoratory of EXcellence Design of Alloy Metals for low-mAss Structure (LabEx DAMAS), Université de Lorraine, Nancy 54011, France; 3 SIMAP, CNRS, Grenoble INP, University of Grenoble Alpes, F-38000 Grenoble, France; 4 UNIROUEN, INSA Rouen, Normandie Université, CNRS, Groupe de Physique des Matériaux F-76000 Rouen, France; raphaele.danoix@univ-rouen.fr (R.D.); peter.jessner@constellium.com (P.J.); beatrice.hannoyer@univ-rouen.fr (B.H.); frederic.danoix@univ-rouen.fr (F.D.); 5 Institut de Chimie de la matière condensée de Bordeaux, University of Bordeaux, UMR5026, F-33600 Pessac, France; mm.goune@gmail.com (M.G.); sylvie.bordere@icmcb.cnrs.fr (S.B.); 6 Matériaux Ingénierie et Science, UMR CNRS 5510, INSA Lyon, University of Lyon, Bât. Saint-Exupéry, 25 Avenue J. Capelle, F-69621 Villeurbanne CEDEX, France; thierry.epicier@insa-lyon.fr

**Keywords:** excess nitrogen, clusters precipitation, Fe–Si and Fe–Cr nitrided alloys, APT and TEM characterization

## Abstract

Atom Probe Tomography (APT), Transmission Electron Microscopy (TEM), and 3D mechanical calculations in complex geometry and anisotropic strain fields were employed to study the role of minor elements in the precipitation process of silicon and chromium nitrides in nitrided Fe–Si and Fe–Cr alloys, respectively. In nitrided Fe–Si alloys, an original sequence of Si_3_N_4_ precipitation was highlighted. Al–N clusters form first and act as nucleation sites for amorphous Si_3_N_4_ nitrides. This novel example of particle-simulated nucleation opens a new way to control Si_3_N_4_ precipitation in Fe–Si alloys. In nitrided Fe–Cr alloys, both the presence of iron in chromium nitrides and excess nitrogen in the ferritic matrix are unquestionably proved. Only a certain part of the so-called excess nitrogen is shown to be explained by the elastic accommodation of the misfit between nitride and the ferritic matrix. The presence of immobile excess nitrogen trapped at interfaces can be highly suspected.

## 1. Introduction

The automotive industry is constantly looking for ways to reduce the weight of vehicles without sacrificing safety, performance, or durability. The development of both high-strength and low-density steels is a timely and appropriate answer to this challenge. In this context, the substitution of carbon by nitrogen in steel metallurgy could provide a path to such high-performance steels with a reduced manufacturing footprint. First, the eutectoid temperature in the binary Fe–N is much lower than in the Fe–C system (592 °C vs. 723 °C) leading to lower heat treatment energy consumption [1]. Second, the strengthening from nitrogen both in solid solution and precipitated in nitrides leads to high mechanical properties [2,3]. Finally, the presence of nitride forming elements can induce a very fine and intense precipitation of nitrides in α-Fe having a density significantly lower than iron, decreasing the density of the final material through composite effect [4]. However, the low solubility of nitrogen in liquid iron (0.046 wt.% at 1600 °C) constitutes however the main barrier to the development of low-alloy nitrogen steel through regular steelmaking [5]. Nitriding treatments offer an interesting alternative in order to mitigate this difficulty. They consist in making nitrogen atoms diffuse from the surface to the bulk into very thick samples and at a given temperature. The diffusing nitrogen then interacts with iron and/or alloying elements to form nitrides. The presence of these nanometre scale nitrides will affect the structural properties such as density, strength, as well as corrosion and wear resistance [6]. These properties depend on precipitation characteristics (nature, volume fraction, density, and mean size of particles), which are themselves influenced by process parameters and both the nature and the amount of alloying elements. For a better control of the nitride precipitation processes, it is important to understand the role of elements present in the alloy. It is expected that iron, which is in even greater quantity, and trace nitride forming elements such as Al, Ti, or V, will strongly affect the precipitation process. This is the main topic of this paper, in which we will focus on two important aspects. On the one hand, the role of aluminium as a minor element on the nucleation of silicon nitrides during nitriding of Fe–Si alloys. On the other, the study of both the iron content of nanometre nitrides and the so-called “excess nitrogen” in nitrided Fe–Cr alloys.

Regarding the precipitation of silicon nitride during nitriding of Fe–Si alloys, this topic has recently received growing attention [7]. Recent works show that the precipitation of silicon nitrides is taking place in the form of cubic and amorphous particles in Fe–Si alloys. The nanometre size and high density of these particles significantly increase the hardness of the alloy, and the low density of silicon nitrides decreases the mass of a structure [6,8]. A transition to crystalline precipitation occurs when excess nitrogen is removed during annealing [4,9]. The origin of such behaviour is still controversial as evidenced by some recent lively discussions in [4,6,7,8,9]. The stability of the amorphous nitride remains an open question all the more so because there are few examples in the literature reporting the formation of an amorphous phase in a crystalline matrix [10,11,12,13]. The purpose of this study is thus to investigate the role of minor alloying elements in the precipitation process of amorphous silicon nitrides in nitrided Fe–Si alloys. To this end, atom probe tomography (APT) was employed. The usual overlap in mass spectra between Si^++^ and N^+^ ions has been overcome by using commercially pure ^15^N.

As for chromium nitride in nitrided Fe–Cr alloys, earlier studies based on atom probe tomography and transmission electron microscopy (TEM) have evidenced that significant levels of iron are present in nitrides [14,15]. However, these results are frequently disregarded because the observed Fe enrichments in CrN can result from experimental artefacts, at least partly. Nevertheless, as will be shown in this paper, recent experimental observations have confirmed the presence of Fe in CrN, even if careful quantitative measurements have shown lower Fe level than initially stated. The consequences of these observations are many. First, if the assumption of stoichiometric MN nitrides (where M stands for metal) is to be ruled out, the volume fraction of particles cannot be simply deduced from a simple lever rule. Unless the amount of iron in the particles is carefully measured, the volume fraction of precipitates remains undetermined. The presence of iron in the precipitates would also modify the thermodynamics of the system and transformation tie-lines. As a result, the kinetics of precipitation, the resulting precipitation state, and mechanical properties of the nitrided layer must be affected and numerical predictions remain inaccurate as long as they do not account for this. Another important potential consequence of the presence of iron in nitrides is the extent of the so-called “nitrogen excess”. In the classical view of MN nitride precipitation, the uptake of nitrogen should be limited to the sum of the amount necessary for precipitation of all alloying elements such as stoichiometric nitrides and the equilibrium nitrogen content in ferrite [16]. However, Fe–Cr has demonstrated a considerable capacity for the uptake of larger amounts of nitrogen than expected [17,18,19]. The additional amount of nitrogen is called excess nitrogen. Keeping the assumption that each metal atom in nitrides binds to one nitrogen atom, that is, nitrides can be considered as (Fe, M)N compounds, the presence of iron in the precipitates leads to an increase in the maximal fraction of nitrides, proportional to the amount of iron they contain. The presence of iron in nitrides could therefore provide a simple explanation to the observation of excess nitrogen as suggested by Ginter et al. [15]. Consequently, it appears essential to confirm the presence of iron in nitrides, and to estimate, as finely as possible, the amount of iron in nitrides, as well as the amount of nitrogen and chromium dissolved in the ferritic matrix (α-matrix). Because of the very fine scale involved, sub-nanometre resolution combined to sensitivity to nitrogen is required. As it fulfils these requirements, APT is the ideal technique for such investigations. Some complementary analyses by analytical TEM (electron energy loss spectrometry, EELS) on two-step extraction replicas are also proposed and reinforce conclusions obtained by APT. The nature of the excess nitrogen uptake, which is a key point in improving our understanding of nitriding treatment, is still matter of debate. In order to address this topic, 3D calculations are performed for determining both the stress field and elastic strain energy induced by the presence of CrN in the α-matrix in complex situations of geometry and anisotropy of elastic properties. The effect of mechanical fields on the nitrogen dissolved in the strained iron lattice is calculated. Comparisons between the measurements and the calculations allow for discussing both the presence and the nature of the so-called excess nitrogen.

## 2. Experimental Procedure

The materials investigated in the present study are ferritic Fe–5at.% Cr and Fe–3at.% Si alloys. Fe–5at.% Cr samples were nitrided in gaseous atmosphere at 590 °C for 12 h (see [20] for details). The internal structure of the diffusion layer is described in details elsewhere [21]. In order to investigate the stability of iron in the chromium nitrides and the nitrogen content in the matrix, a second set of specimens was prepared. It received an additional thermal treatment of 24 h at 590 °C in vacuum. 

Fe–3at.% Si samples were rolled into sheets 1 mm in thickness and plasma nitrided at 570 °C for 1 h using commercially pure ^15^N in order to avoid the usual overlap in mass spectra between Si^++^ and N^+^ ions at 14Da during atom probe analyses. Details of the method can be found in [4]. Both sides of the sheets were nitrided. Afterwards, samples were annealed at 570 °C in vacuum (10^−6^ mbar) for 20 h.

Depending on the material, and the thickness of the nitrided layers, the specimens for atom probe tomography analyses were prepared combining mechanical grinding and electrochemical etching for the Fe–Cr alloy [20], and focussed ion beam (FIB) lift out and annular milling for the Fe–Si one [22]. Each sample was finished to a tip radius smaller than 50 nm. Field ion microscopy (FIM) (Home built, University of Rouen, Rouen, France) was conducted at a temperature of 80 K and voltages between 10 and 15 kV, using neon as imaging gas. APT measurements on the Fe–Cr alloy were performed at 80 K on a CAMECA energy compensated tomographic atom probe (ECOTAP), equipped with an advanced delay line detector [23], with a pulse repetition rate of 2 kHz and a pulse fraction of 20%. Three-dimensional reconstructions were obtained using the software (Home built, University of Rouen) developed at the University of Rouen, based on the algorithm described in [24]. Concerning the Fe–Si alloy, analyses were conducted on a Cameca LEAP^®^ 4000HR (Cameca Inc., Madison, WI, USA), using the same experimental conditions, except for the pulse repetition rate, which was increased to 200 kHz. Three-dimensional reconstructions were obtained using Cameca IVAS^®^ 3.6.8 (Cameca Inc., Madison, WI, USA).

The determination of the composition of the precipitates was conducted using a cluster identification method. Threshold concentrations in one or more elements for the phase under consideration are defined. For each atom, the local concentrations in a surrounding sphere of radius 1 nm are calculated, and compared to the threshold values. If the measured concentrations lie within the given limits, the tested atom is considered as being part of a so-called cluster [25,26].

Specimens for TEM investigations were prepared using the two-step extraction replica method [27]. As the iron concentration in nitrides is of interest, any contribution to the determination of the nitride composition arising from the ferritic matrix needs to be eliminated. It is therefore not possible to measure the nitride concentration directly from thin foils. Therefore, extraction replicas on carbon films have to be fabricated from the nitrided Fe–5at.% Cr alloy to meet these requirements. As the region of interest is the diffusion layer, the surface area of a bulk specimen, with a section of 5 × 5 mm^2^, has been removed by grinding and polishing the sample to a depth of 100 μm from the original surface. For extraction replicas, the sample surface is further attacked with Nital 2%. A cellulose-acetate polymer film was glued on the surface and pulled off. Nitride precipitates stick on the polymer-film on which a carbon film, approximately 50 nm thick, is deposited. Subsequently, the polymer is dissolved in a solution of methanol in dichloromethane and the extracted nitrides embedded in the carbon film are recovered on TEM grids. By applying this two-step technique, a possible contamination of iron during the sample preparation process is minimized, as will be discussed below. Electron energy-loss spectrometry (EELS) was performed on a field emission gun JEOL 2010F electron microscope operated at 200 kV. The microscope is equipped with a Gatan DigiPEELS spectrometer with a standard photodiode array detector. EELS spectra were recorded with the specimen at room temperature using the following parameters: Convergent half-angle α = 10°, collection half-angle β = 7°, acquisition time equal to 10 s, probe size focused to about 3 nm in TEM mode, and a nominal energy dispersion of 0.5 eV. The experimental spectra were classically corrected from the dark current and channel-to-channel gain variations of the detector, and then de-convoluted from the multiple scattering using the Fourier-ratio technique. The background under each edge ‘*i*’ (where *i* stands for N, Cr, Fe) of interest is subtracted according to its extrapolation from a power-law, and fitted over an optimized energy window ∆E*_i_* preceding each edge. According to the elements present (Fe, Cr, N), the chemical analysis can be conducted using the classical method proposed by Egerton [28]. To evaluate the accuracy of the measurements, the energy windows used have been optimized on standard spectra CrN and Cr_2_N used by Mitterbauer [29]. An accuracy of at least about 5% can be obtained for the atomic ratio [Cr]/[N]. This accuracy is of the same order of magnitude as demonstrated by Courtois in the case of NbCN precipitates examined on extraction replicas [30].

TEM thin foils were prepared from disks of 1.5 mm in radius machined at a distance of about 100 μm from the surface. These disks were electrochemically thinned in a Struers Tenupol at 27 V around 10 °C and cleaned by using a plasma system. They were mainly observed in a cold FEG JEOL ARM 200F and additional observations were carried out in a Philips CM12 (Philips-FEI, Hillsboro, OR, USA) a Philips CM200 (Philips-FEI, Hillsboro, OR, USA) and a JEOL 2010F.

## 3. Calculation of the Elastic Stresses and Strain Energies

The mechanical calculations were performed using the THETIS code [31]. The simulation system is a cube of representing the ferritic matrix. Periodic conditions and an external pressure *p* = 0 were used to allow the system to relax the anisotropically deformed particle. The pressure p corresponding to the spherical part of the stress tensor ***σ*** and the shear stress tensor ***τ*** were considered. The relationship between ***σ*** and ***τ*** is given by:

(1) σ=−pI+τ

Both the stress and strain fields induced by the formation of the nitride particles into the ferritic matrix were then calculated. The total elastic strain energy density stored into the system was determined from:

(2) $$G^{el,\Phi }=\frac{1}{V_{t}}\int_{V_{t}}\frac{1}{2}\epsilon :\sigma dV$$

where ***ϵ*** is the strain tensor and *V_t_* is the total volume of the system.

## 4. Results and Discussion

### 4.1. Mechanism of Amorphous Si_3_N_4_ Precipitation in Nitrided Fe–Si Alloys

**Figure 1:**
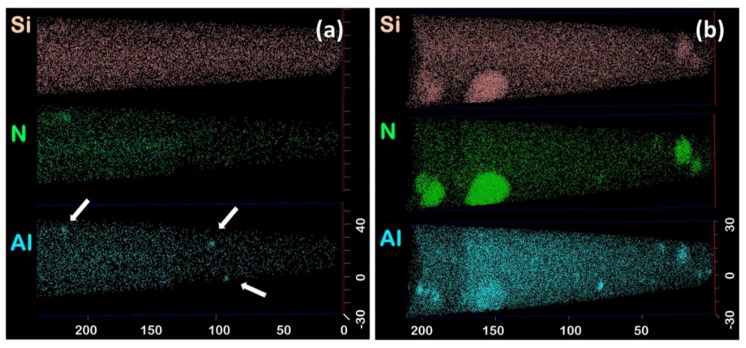
Tomograms of Si, ^15^N, and Al in (**a**) the as-nitrided sample and (**b**) after 20 h of annealing. The arrows indicate Al–N clusters. Only 50% of detected Si atoms are shown for clarity (Scale in nm).

Atom probe analyses were carried out on four specimens sampled from the as-nitrided Fe–Si alloy, the typical volume of which was 50 × 50 × 200 nm^3^. One such volume shown in Figure 1a was found to be representative of all other volumes. The chemical composition of the alloy based on the four analyses is reported in Table 1. The concentration in Si, which is the major alloying element, was found to be very close to the nominal one (3.1 at.%). The concentration in residual Al, Ti, and Mn are about 0.06, 0.03, and 0.01 at.%, respectively. Traces of C were also detected.

On the one hand, frequency distribution analysis confirmed that Si is homogeneously distributed. Added to the fact that Si concentration in the matrix is close to the bulk concentration, it suggests that both the fraction and number density of silicon nitride are very low, if non-zero, in the as-nitrided condition. On the other hand, residual Al was found to form Al–N clusters that are visible with the naked eye on tomograms, as illustrated in Figure 1a, where they are highlighted with arrows. These clusters were also systematically identified and delineated using 7 at.% Al isosurfaces. Local magnification, a technique-related artefact that causes incorrect positioning of atoms in the immediate vicinity of phase interfaces, prevents any accurate calculation of the exact composition of such small clusters [32,33]. However, their Al/N ratio proved to be roughly 1.1, close to the theoretical ratio of Al/N.

Figure 1b shows the tomogram of a typical atom probe specimen in the annealed condition, in which silicon nitride precipitates are visible. It exhibited lower matrix concentrations in Si, Al, and N. This drop in concentration can be ascribed to the precipitation of the observed silicon nitride. After correction for the overlap of ^30^Si/^15^N and ^28^Si/^14^N (residual), Si and N contents in the precipitates was determined to be ≈42 at.% and ≈55.5 at.%, respectively, leading to a Si/N ratio of 0.757. This ratio is in excellent agreement with the theoretical ratio of Si_3_N_4_, suggesting that the precipitates deviate little from stoichiometry, which confirms similar reports based on different techniques [4,7,9]. Al, Ti, and Mn were found to be present in the precipitates at contents of 1.75, 0.27, and 0.14 at.%, respectively. The concentrations of the precipitates in these elements were found to vary considerably, up to about 40 at.% in the analysed particles, probably due to locally different precipitation states. The residual Fe found in the precipitates is likely to be marginally overestimated due the possible ^28^Si^+^/^56^Fe^2+^ and ^14^(N_2_)^+^/^56^Fe^2+^ peak overlaps and/or trajectory overlap of ions [34].

**Figure 2:**
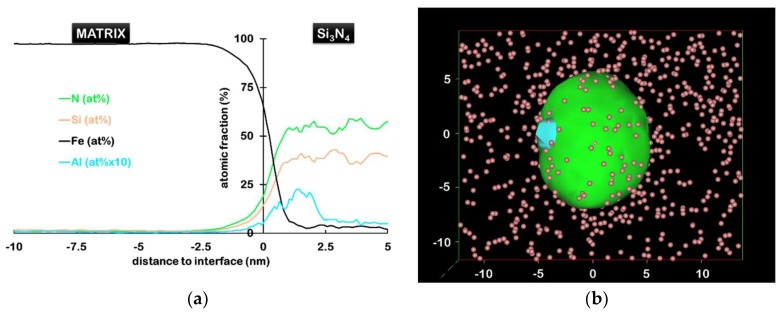
(**a**) Proxigram across Si_3_N_4_/matrix interfaces shown in (**b**). Al–N clusters, selected by 7 at.% isosurface (blue), can be seen at the interface between the Si_3_N_4_ precipitate (isosurface N = 25 at.%, shown in green) and matrix (**b**). For clarity, only Si ions in the matrix are shown.

Based on silicon nitride precipitates intercepted in six atom probe specimens, their number density could be estimated to about 10^21^ m^−3^. Clustering of Al and N atoms is obviously more pronounced here. It is noteworthy that all Si_3_N_4_ are associated with an Al rich region, but not reciprocally. Al–N clusters were found to be more numerous than Si_3_N_4_ precipitates, at about 4 × 10^21^ m^−3^, which is still the same order of magnitude. Since they are also anterior to the silicon nitride precipitates, it suggests that they act as nucleation sites for Si_3_N_4_. The proximity histogram (proxigram) shown in Figure 2a, is constructed based on a 25 at.% N isosurface, by calculating concentration as a function of the distance to this interface, from the tomogram shown in Figure 1b [36]. It indicates that silicon nitride precipitates are enriched in Al (mostly at the interface) as well as in Ti and Mn (not shown here). However, since the concentrations are averaged over multiple precipitates, it does not represent accurately the distribution of elements in each individual precipitate. In particular, it has been observed in Figure 2b that Al–N clusters can lie at the interface between the Si_3_N_4_ precipitates and the matrix.

These results indicate first that the formation of Si_3_N_4_ precipitates occurs after the formation of Al–N clusters. This is consistent with the high affinity of Al with N and the large volume misfit reported for Si_3_N_4_ that results in its high nucleation energy barrier [8]. Further, the fact that every observed Si_3_N_4_ precipitate contain, or is directly adjacent to, Al–N clusters suggests they nucleated heterogeneously at the interface of these clusters. This is once again consistent with too high a homogeneous nucleation barrier leading to heterogeneous precipitation on low energy sites. This has already been observed on dislocations [8], and for other nitrides [37] on Al–N clusters as well. Consequently, the number density of Si_3_N_4_ nucleation sites is directly related to the number density of those clusters, a point that is often disregarded for simplicity’s sake in classical precipitation models, where each substitutional is assumed to be a potential nucleation site [9,38]. The present result shows that this approximation does not hold in the case of silicon nitride and opens new strategies in controlling silicon nitride precipitation.

### 4.2. Experimental Evidence of Iron in CrN in Nitrided Fe–Cr

#### 4.2.1. Field Ion Microscopy & Transmission Electron Microscopy

The typical chromium nitride precipitates, formed in the diffusion layer of nitrided Fe–Cr alloys are thin disc-shaped platelets with nanometric dimensions [21,39,40,41]. They exhibit a face centred cubic structure, with the metal atom on the fcc sites and nitrogen occupying the octahedral sites. The [100] direction of the nitride is then parallel to the [110] direction of the bcc matrix, with a misfit of about 2.34%. In the perpendicular direction, the misfit is much larger, close to 45% [42]. Therefore, coherent interfaces along the (001) planes and incoherent interfaces along the (100) and (010) planes develop. The growth rate in the direction normal to the incoherent interface is much faster than in the direction of the coherent interfaces [43]. The latter are forced to migrate by a ledge mechanism, which explains the disk-shaped form of the nitrides. Due to symmetries in the cubic system, three variants of platelets develop, with habit planes lying in the bcc {001} planes.

**Figure 3:**
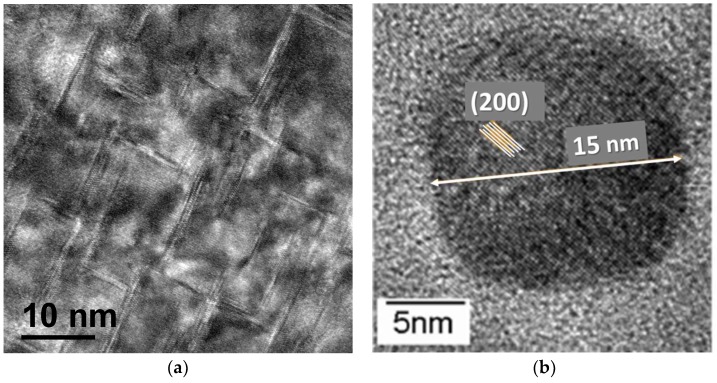
(**a**) High-resolution transmission electron microscopy (HR-TEM) micrograph recorded along the [001] zone axis. In this configuration, only two variants are visible, the third, lying parallel to the surface, is hidden; (**b**) High-resolution view on a single nitride obtained from a replica, with (200) fcc planes in the precipitate indicated.

These platelets can be observed by high-resolution transmission electron microscopy (HR-TEM), provided the thin foil is observed along <001> zone axis. On the HR-TEM, recorded along [001] axis, only two visible variants are developed along [100] and [010] directions, respectively. The third variant is revealed edge-on in the HR-TEM micrograph recorded along [001] zone axis, in Figure 3b.

**Figure 4:**
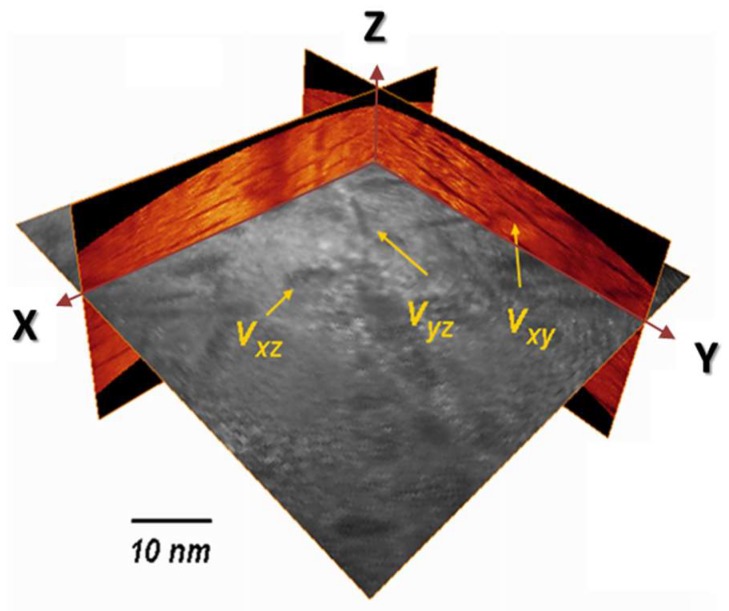
3D-FIM reconstruction of analysed volume in the nitrided layer, showing the three variants of CrN platelets. CrN platelets appear in dark contrast. The different variants, referred as *Vxy*, *Vyz* and *Vxz* are indicated with yellow arrows.

The same microstructure was investigated using three-dimensional field ion microscopy (3D-FIM) to reveal the three variants simultaneously. Details related to 3D-FIM can be found in [44,45]. Figure 4 shows three sections of the analysed volume: Two along the analysis direction (in orange) and one perpendicular (in black and white). The three variants of CrN appear in dark contrast for all sections.

Combining HR TEM and 3D-FIM, the average diameter of CrN platelets is found to be close to 20 nm, and their thickness about 1 to 2 nm.

#### 4.2.2. Atom Probe Tomography

**Figure 5:**
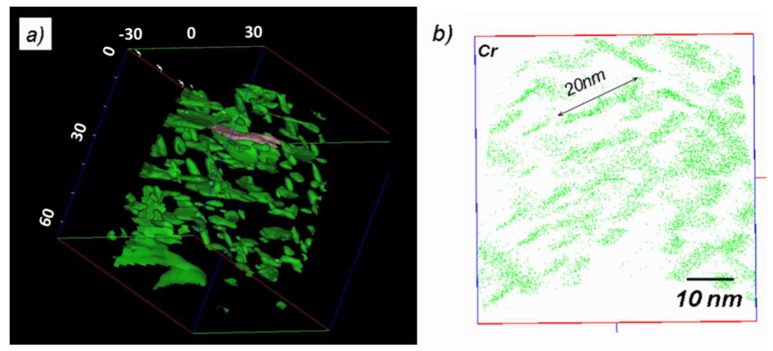
APT reconstruction in the nitrided layer containing CrN: (**a**) isoconcentration Cr + N > 40 at.%, showing the morphology and distribution of CrN platelets; (**b**) atom map showing the location of individual Cr atoms. For clarity, the represented volume is restricted to a 2 nm thick slice.

A typical APT reconstruction is shown in Figure 5a. Due to the high density of precipitates, standard atom maps are difficult to interpret, and the nanostructure is better revealed using isoconcentration surfaces. The green surfaces in Figure 3a are surrounding regions where the local Cr + N content is higher than 40 at.%. They clearly correspond to CrN particles. In this reconstruction, the three variants can be observed, and the average dimensions are coherent with those previously derived, as shown in the atom map in Figure 5b.

**Figure 6:**
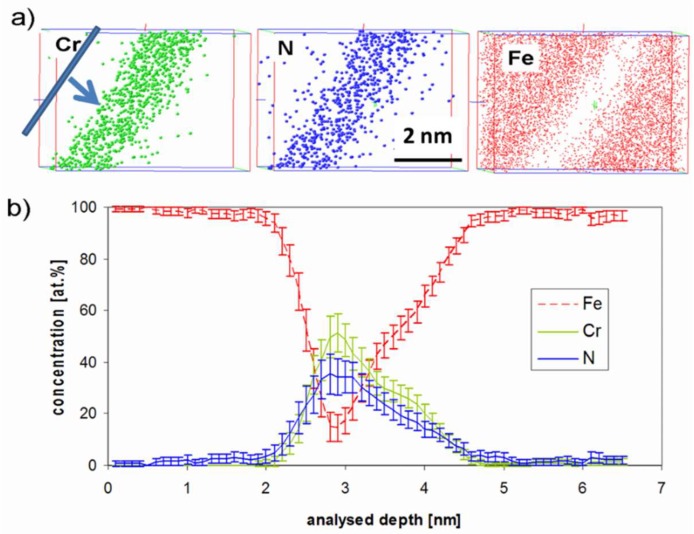
(**a**) Elemental atom maps around a selected nitride and (**b**) corresponding composition profile obtained perpendicular to the nitride habit plane, with a resolution of 0.1 nm. The sampling volume (0.1 nm thick) and profile direction are indicated on the top left of the Figure 6a.

Concentration profiles are plotted by moving sampling sub-volumes along a given direction. The main advantage of this technique is that the spatial resolution of the profile is defined by the operator (sampling volume thickness), and that the profile direction can be chosen perpendicular to the nitride habit planes, whatever the orientation of the specimen. Together with a lateral extension of the sampling volume smaller than the nitride diameter, concentration profiles with minimized spatial overlaps between matrix and precipitates are obtained, and nitride composition obtained with the highest accuracy. This procedure is illustrated in Figure 6.

The concentration profile shown in Figure 6b indicates that there is 15 at.% of residual iron, still present in the core region of the nitride.

**Figure 7:**
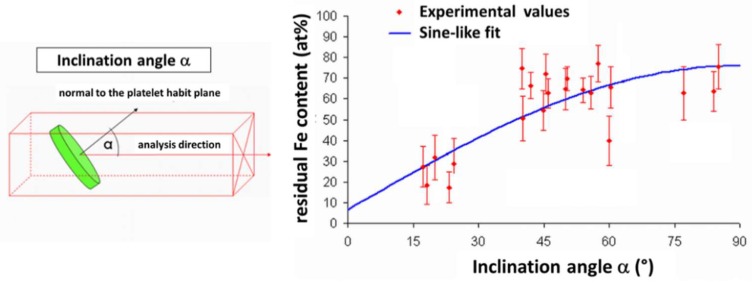
Measured Fe-concentration against the inclination angle between nitride and analysis direction.

Because of the experimental artefact of local magnification, this value should be used with caution. Indeed, it is known that the local composition in thin layers (such as platelets in this case) determined by APT depends on the angle between the layer and analysis direction [46]. This is due to a potential overlap of ion trajectories in the close vicinity of the matrix-platelet interfaces, caused by a difference in evaporation field between the two phases, known as local magnification. When the evaporation field is higher in the matrix, ions initially in this phase can erroneously be positioned in the platelets in 3D reconstructions. This effect is absent when the platelet is perpendicular to the analysis direction (inclination angle equal to zero), and increases with the inclination angle. As previously mentioned, nitrides appear in dark contrast in FIM micrographs, indicating that they have a lower evaporation field (or cohesive energy) than the matrix. Consequently, it is expected that the amount of residual iron in the nitride will depend on their orientation with respect to the analysis direction. In order to investigate this effect, the apparent iron content in nitrides has been determined as a function of the inclination angle. Figure 7 reports this evolution, as obtained from different samples having various orientations.

The iron content apparently increases with the inclination angle from 0 to 90°, which is consistent with the local magnification effect. For geometrical reasons [21], the experimental data are fitted by a sine-like function, and the best fit is obtained by a least squares method. The extrapolation of the fit to the zero-inclination angle (where artificial mixing is supposed to be absent) leads to a value of 6.6 at.% Fe, considered as the actual residual amount of Fe in the CrN platelets.

It is worth noting that a nitrogen deficit is always observed during the APT analysis of CrN, leading to a Cr/N ratio smaller than 1. Based on the hypothesis that this deficit is mostly due to evaporation artefacts, the real nitrogen concentration in the nitride is higher than measured by APT, and thus the iron concentration measured has to be seen as an upper limit.

In order to confirm this result based on sine-like fit of the experimental data, the use of a complementary technique is necessary. Therefore, analytical TEM investigations were conducted to independently measure the iron content in nitrides.

#### 4.2.3. Analytical Transmission Electron Microscopy

Previous TEM investigations on nitrides in nitrided systems have been performed on one-step extraction replicas [15,22,47,48]. The preparation technique of these replicas can result in an incomplete dissolution of the ferritic matrix surrounding the nitride particles. Such matrix residue would later produce an unwanted iron signal during TEM analyses. Two-step replica extraction was therefore preferred here, being less likely to produce this artefact. To the knowledge of the authors, this is the first attempt at quantifying iron in chromium nitride using this preparation technique.

**Figure 8:**
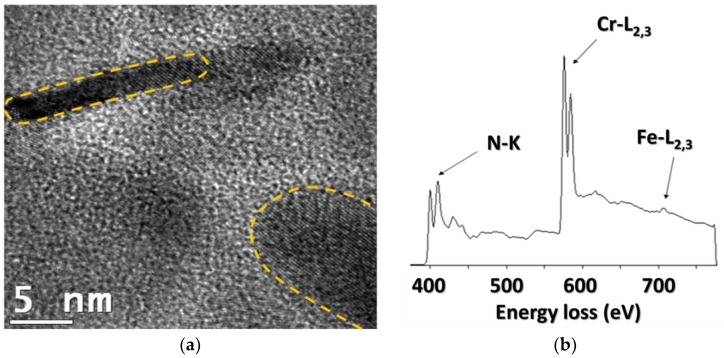
(**a**) TEM micrograph of nitride replicas on carbon film and (**b**) corresponding electron energy loss spectrometry (EELS) spectrum indicating the presence of Fe in Cr-rich nitrides.

The micrograph in Figure 8 shows a group of nitrides on the supporting carbon layer with the corresponding EELS spectrum. Two nitrides, one upright and one horizontal, are marked by dashed lines, indicating their disk-shaped form. They show a diameter of approximately 20 to 30 nm, but their thickness is difficult to access, because their exact orientation on the replica is not known. The EELS spectrum exhibits the ‘saw tooth’ edge of nitrogen (N-K) and the white lines of the chromium L_2–3_ edge. The iron peak Fe-L_2,3_ is low, but still emerges from the background after the Cr peak. Applying the analytical method proposed by Egerton [28], the ratios of Fe/Cr and Cr/N have been determined to be Cr/N = 0.9 and Fe/Cr = 0.14. This leads to a ratio of (Cr + Fe)/N = 1.03. Both the platelet shaped form and the stoichiometry are consistent with fcc-NaCl-type CrN nitrides. The accuracy of the measurements is estimated to be approximately 5%.

**Figure 9:**
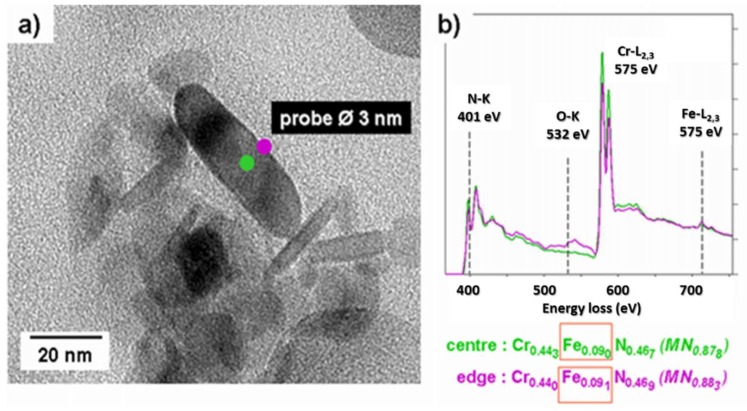
EELS spectra (**b**) of a nitride lying on a carbon replica **(a**) and corresponding Cr and Fe contents. (The Fe-concentration does not differ significantly between the core of the nitride and its edge).

The key point here is to identify the location of detected Fe-atoms: Are they incorporated in the nitrides, or concentrated at their surfaces, due to the replica extraction technique. If iron residues were mostly deposited on the particle surfaces, this would lead to a more pronounced iron concentration along the edges of the particles than in their centres, as the surface over volume ratio would be larger. Figure 9b shows the EELS spectra obtained in both regions of the same particle. The Cr and Fe contents are, within experimental errors, identical in the edge and the core of the particle, strongly indicating that surface contamination can be ruled out. It is concluded that Fe atoms are homogeneously incorporated in the nitrides, partially substituting to chromium, in agreement with previously reported results [49].

This procedure has been reproduced for a total of 44 measurements. The mean chemical composition of the nitride precipitates obtained is (Cr_0.89±0.02_Fe_0.11±0.01_)N_0.88±0.02_, leading to an average residual amount of iron equal to 5.85 at.%.

This result is fully coherent with the value obtained by APT, where 6.6 at.% was considered as the upper estimate. It can therefore be concluded that two independent measurement lead to the same conclusion, that there is about 6 at.% Fe in the chromium nitrides after nitriding.

#### 4.2.4. Equilibrium Concentration of Nitrides

If the previous results prove the presence of iron in CrN right after nitriding, its origin is not clear: Is this presence related to a kinetic effect (slow redistribution of iron) or due to the thermodynamics of this ternary system. In order to elucidate this point, an additional heat treatment, consisting in a simple thermal holding was applied to the as-nitrided specimens. The samples were thus aged for 24 h at 590 °C (the nitriding temperature).

**Figure 10:**
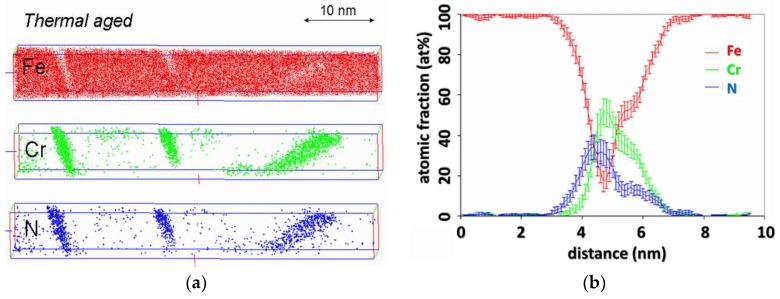
(**a**) Atom probe tomography (APT) tomogram and (**b**) corresponding composition profile (**b**) of nitride platelet in nitrided Fe-5at.% Cr alloy followed by thermal treatment in vacuum for 24 h at 590 °C.

Figure 10a shows tomograms obtained after this further thermal treatment. They clearly show that the nanostructure has coarsened, that is, precipitates have grown (mostly in their habit planes), and their number density has significantly dropped. Figure 10b shows the concentration profiles obtained with the same protocol as previously described on the first nitride on the left of Figure 10a. These concentration profiles clearly indicate that iron is still present in the core of the nitrides. This presence cannot be solely explained by the local magnification effect, as the amount of Fe is similar to the one observed in the as nitrided precipitates, for the same inclination angle (here 15°).

This observation leads to the conclusion that the presence of iron in CrN cannot be regarded as a kinetic effect, but rather as a thermodynamic one. It must be accepted that the equilibrium composition of CrN nitrides includes iron, to a level of approximately 6 at.%.

The classical assumption is that only stoichiometric CrN nitrides are formed, excluding any solvent content in the particles. The reason for such a simplifying assumption is obvious, as it reduces the thermodynamic quantities to be assessed to well-known compounds. Driving forces (equilibrium and kinetic) may be calculated from thermodynamic data related to CrN, as implemented in THERMOCALC^®^. In the ternary Fe–Cr–N system, these data come mainly from the thermodynamic evaluation of the Cr–N and the Fe–N systems [50]. Classically, the formula used for the CrN nitride is Cr_1_(N, Va)_1_ (where Va is vacancies in the second sublattice). It is supposed that only a very small amount of vacancies is allowed on the second sublattice.

The assessment proposed by Frisk [50] supposes that the CrN phase has a very narrow existence range and implies that CrN is considered as a stoichiometric phase.

Therefore, the thermodynamic databases presuppose that the compound formed is stoichiometric and contains no iron. The elastic effects are also neglected. The consequences are twofold. On the one hand, it leads to a distorted description of the system thermodynamics, because the precipitate equilibrium volume fraction is skewed. On the other hand, since thermodynamics and kinetics are coupled, it also affects the description of precipitation kinetics through the evaluation of the driving force for both nucleation and growth. More precisely, the tie lines, which depend on thermodynamics and kinetics, will be affected. A consequence of the present finding is that it may potentially question the classical thermodynamic treatment of nitriding, not only in Fe–Cr, but also in all the systems where nitrides potentially contain iron.

Recently, thermodynamic calculations performed with Thermocalc software show that chromium nitrides can incorporate up to 22 at.% of iron during nitriding of Fe–3at.% Cr alloy at 520 °C [15]. Regarding the nature of the presence of iron in chromium nitrides, it may be addressed in two ways. First, iron is the solvent, and is therefore present in very high quantities in the base material. Second, iron nitride FeN has a lower solubility product constant than chromium nitride CrN. A simplistic description of a mixed nitride in the framework of the ideal solid solution as proposed by Hillert and Staffansson [51] gives additional clarifications. Indeed, the mixed nitride (Fe, Cr)N can be regarded as a combination of the two nitrides CrN and FeN. With the regular solid solution model, the amount of iron in the nitride particles depends on the amount of iron in the matrix, and the relative stability of FeN with respect to CrN. Thermodynamic data show that iron nitride is 10 times more stable than CrN at the nitriding temperature [52]. The calculations performed by this approach explain the presence of iron in particles (from 5% to 95% of iron), with the content of iron depending on the solubility product of FeN with respect to CrN [21]. Consequently, the iron content in CrN precipitate that was measured experimentally is consistent with this modelling approach.

### 4.3. Excess Nitrogen in Fe–Cr–N System

#### 4.3.1. Effects of Iron

Some systems (Fe–V, Fe–Cr) have a considerable capacity for the uptake of so-called excess nitrogen. Indeed, it was observed that the nitrogen uptake was larger than necessary for simultaneously precipitating the entire alloying element content as stoichiometric nitride and reaching the equilibrium nitrogen content in the ferritic matrix. The origin of that behaviour is not fully understood but it can be suspected that the presence of iron in the corresponding nitride (CrN in our case) could explain or influence the observed excess nitrogen. Indeed, it can be supposed that substituting Fe to Cr in CrN would occur at a constant M/N ratio. This would result in a Cr/N ratio lower than 1 and iron would thus contribute to nitrogen excess. In other words, if the Cr/N ratio does not change, it means that iron in nitrides cannot explain excess nitrogen, either in whole or in part.

As our experimental results show that the Cr/N ratio is never smaller than 1, (equal to 1 in the case of EELS analysis), it can be concluded that, contrary to what had been suggested [15], the presence of iron in chromium nitrides does not contribute to excess nitrogen in nitrided Fe–Cr alloys.

#### 4.3.2. Effect of Elastic Accommodation of the Misfit between Nitride and Matrix

**Figure 11:**
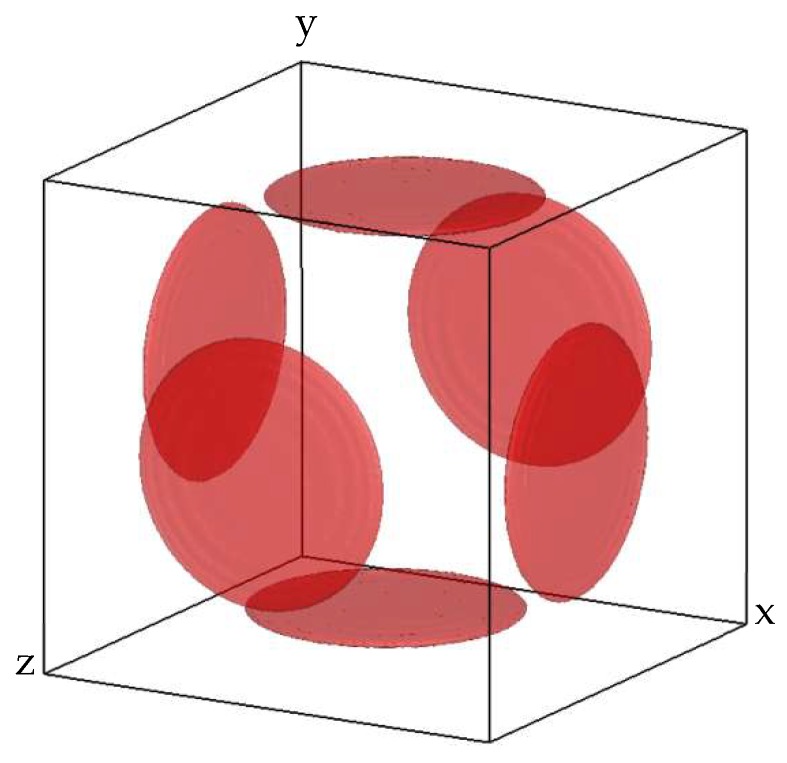
Representation of the 3D configuration of chromium nitride platelets at a volume fraction of 5%.

TEM and APT analyses show that nitrides grow along three orthogonal directions of space with a disk morphology. A 3D representation of this configuration corresponding to a 5% volume fraction is shown in Figure 11.

The initial strain of the platelets was obtained from the lattice mismatch between CrN and ferrite based on electron diffraction patterns. The anisotropic elastic strain related to the morphology of the particles can be written as the strain tensor below:

(3) $$\varepsilon =\left(\begin{array}{ccc} \varepsilon_{E} & 0 & 0\\ 0 & \varepsilon_{L1} & 0\\ 0 & 0 & \varepsilon_{L2} \end{array}\right)$$

with $\varepsilon_{E}=-O.45$ in the thickness direction and $\varepsilon_{L1}=\varepsilon_{L2}=-0.023$ in the main axes of particles. These data are fully coherent with the misfits measured along the [100] and [110] directions.

The properties of the nitrides and the matrix used for the mechanical calculations are summarized in Table 2.

**Figure 12:**
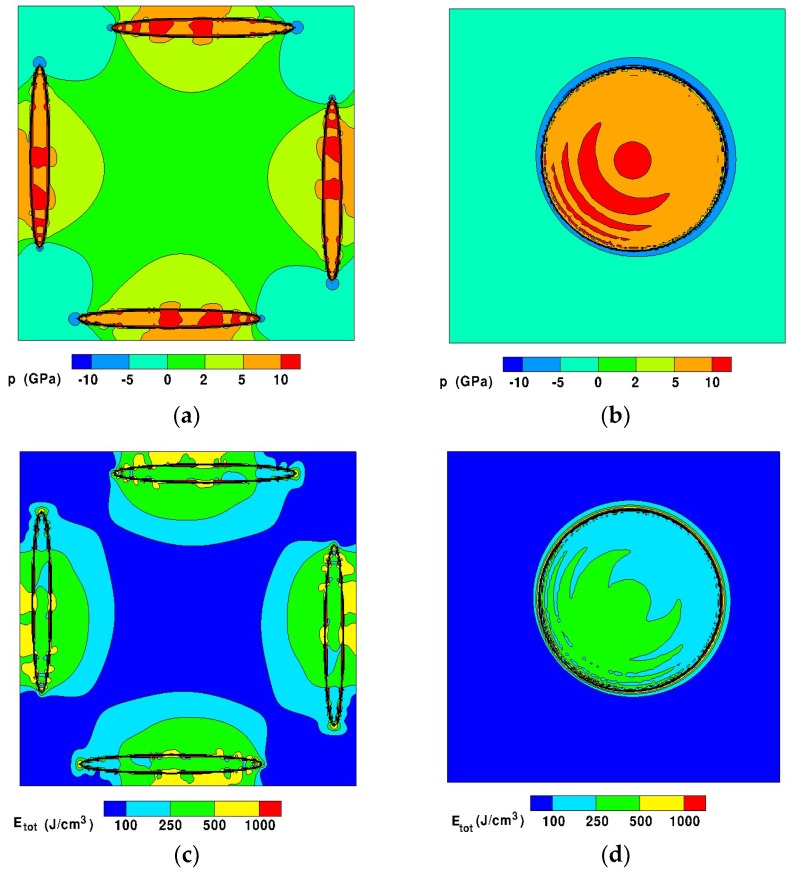
Results of mechanical calculations for a 5% volume fraction of precipitates. (**a**) Pressure map in plane (X = 0.5; Y; Z); (**b**) Pressure map in plane (X = 0.065; Y; Z); (**c**) Total elastic strain energy map in plane (X = 0.5; Y; Z); (**d**) Total elastic strain energy map in plane (X = 0.065; Y; Z).

The calculation of the initial elastic strain relaxation, leads to the equilibrium energy configuration that can be visualized as stress and strain energy distribution maps. For example, pressure and total elastic strain energy in characteristic planes of the system for a precipitate volume fraction of 5% are shown in Figure 12.

Overall, it can be noted that the matrix is under strong pressure gradients, from compression in the centre of the system to tension at the edges. As expected, the particles are strongly compressed.

In order to determine the influence of the stress field on the thermodynamic equilibrium, the evolution of the elastic energy stored in the system can be calculated as a function of the volume (or atomic) fraction of precipitates. In that case, from the work of [53] and if the surface tension contribution is neglected, it can be shown that the solubility product is affected in the following manner [54]:

(4) $$K_{el}^{\Phi }=K^{\Phi }exp\left(\frac{V^{\Phi }}{RT}\frac{\partial G^{el,\Phi }}{\partial f^{\Phi }}\right)$$

where *G^el,Φ^* corresponds to the volumetric elastic energy density in the system due to the formation of phase *Φ* in the ferritic matrix and $V^{\Phi }$ is the molar volume of nitride.

In the same way as for the Gibbs-Thomson effect [55], an additional term to the chemical potential of precipitate, equal to $V^{\Phi }\frac{\partial G^{el,\Phi }}{\partial f^{\Phi }}$, must be added to account for the elastic strain energy stored in the system.

**Figure 13:**
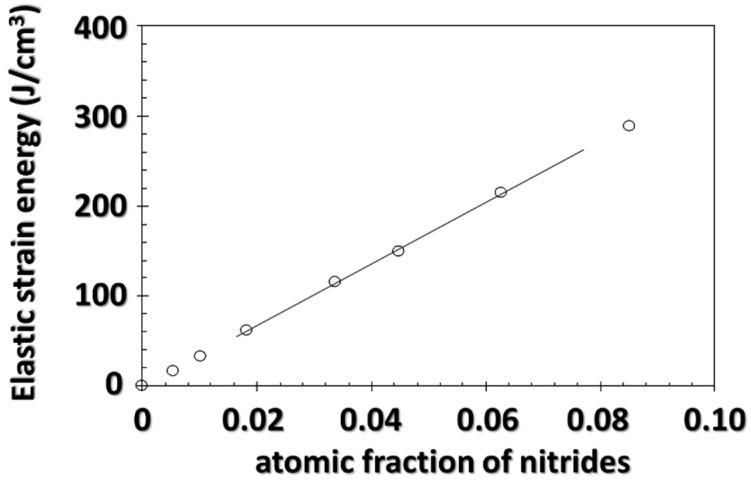
Evolution of the stored elastic strain energy as a function of the atomic fraction of nitrides, based on mechanical calculations.

These mechanical calculations lead to the results shown in Figure 13, where the elastic strain energy is plotted as a function of the atomic fraction of precipitates. To that end, the volume fraction of particles was converted into an atomic fraction.

It should be noted that the first derivative of this curve $\frac{\partial G^{el,\Phi }}{\partial f^{\Phi }}$ is always positive, which indicates that the stress field resulting from the misfit between the particle and its matrix causes an increase in its solubility, in accordance with Equation (4). For atomic fractions below 8% (volume fractions below 10%), the elastic strain energy changes linearly with the atomic fraction of precipitates, with a slope of 3300 J·cm^−3^. This type of evolution has already been observed in the case of coherent equilibria if one assumes a low precipitate fraction and that the elastic properties of the matrix and the precipitate are similar, homogeneous and isotropic. Three important observations should be made about this analysis. First, in a ternary system, it is not only the equilibrium concentration of nitrogen that is affected but also the product of the activities of both chromium and nitrogen, contrary to what is commonly stated [19,56]. Second, as the elastic strain energy changes linearly with atomic fraction of precipitates in the considered range, the increase in nitrogen content induced by the stress field is not expected to be related to the volume fraction of precipitates (see Equation (4)). Finally, this increase cannot be ascribed to the Gibbs-Thomson effect, which can only cause a maximum increase—for precipitate radius the order of interplanar distances—of the solubility product by a factor of 20.

**Figure 14:**
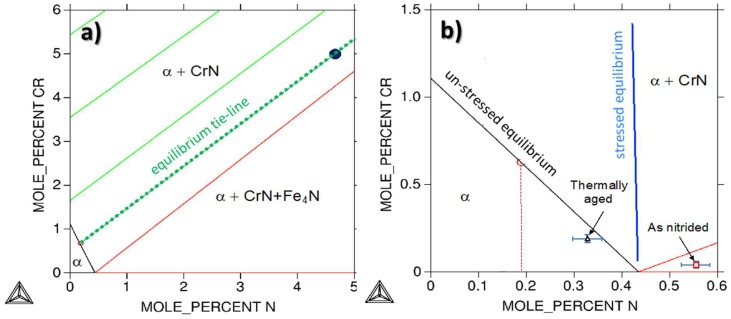
Isothermal sections of the ternary Fe–Cr–N phase diagram at 590 °C. (**a**) Nitrogen and chromium contents in the ferritic matrix in equilibrium with CrN are set by the tie-line running through the alloy composition. (**b**) The solvi of CrN in ferrite, under stress and stress-free, are represented along with nitrogen and chromium concentrations in ferrite after nitriding and after annealing in vacuum for 24 h at 590 °C.

Further analyses can be formulated by comparing the above results to the isothermal section at 590 °C of the Fe–Cr–N ternary diagram, shown in Figure 14. It can first be seen that the composition of the alloy, represented in Figure 14a by a black dot, lies in the two-phase α + CrN region. The nitrogen and chromium contents in the matrix in equilibrium with CrN are set by the tie-line running through the alloy composition as illustrated in Figure 14a. They are found to be 0.18 at.% and 0.61 at.% for N and Cr, respectively. The nitrogen content measured in the matrix is found to be 0.55 ± 0.02 at.%, or three times the predicted equilibrium content. The solvi of CrN with and without the stress field are shown in the iron rich corner of the section in Figure 14b. From this diagram, it is clear that the stress field increases the solubility of CrN in α and thus enlarges the single phase α region. In the presence of the stress field, the solvus is almost a vertical line. However, the predicted N content under stress is 0.43 at.% at most, which is still lower than the measured content. It is reminded here that the calculated solvus under stress is an upper limit of the solubility increase due to elastic strain, as it assumes that the elastic stress is entirely relaxed through equilibrium shift. In reality, only a certain part of stress can be relaxed by modification of the equilibrium state. According to [57], many mechanisms of stress relaxation can co-exist simultaneously. As an example, one can mention crystal shape optimization and plastic deformation. Therefore, the measured excess nitrogen can only be partially explained by stress relaxation. After annealing for 24 h at 590 °C in vacuum, the nitrogen content in the matrix decreases from 0.55 at.% to 0.32 at.%. Part of the mobile nitrogen leaves the sample during the heat treatment. It is interesting to observe that the matrix concentrations now lie closer to the stress-free solvus. However, they do not match the point given by the tie-line corresponding to the alloy, suggesting the presence of immobile excess nitrogen trapped at interfaces.

## 5. Conclusions

In summary, advanced local analysis techniques such as APT and TEM-EELS have been used in the present work to study original aspects of the precipitation of alloying element nitrides in iron based systems during nitriding. Their high analytical resolution at the nanometer scale or below provides invaluable information regarding the precipitation of such nitrides and brings new information regarding issues that had been identified years ago, such as excess nitrogen in Fe–Cr.

In the case of Fe–Si alloys, APT analyses showed, early during the treatment, nitrogen clusters with trace elements with which it has a greater affinity, Al here. As the treatment progresses, stoichiometric Si_3_N_4_ precipitates systematically nucleate on those small but numerous clusters as they provide lower energy barrier sites. As a result, controlling the distribution of those clusters by changing the clustering element or its content, or by using a clustering treatment at a different temperature, should permit, in accordance with the classical precipitation theory, tailoring of the population of subsequent silicon nitride precipitates.

In the case of Fe–Cr alloys, independent measurements using APT and TEM-EELS confirmed the presence of iron in CrN particles precipitated during nitriding at 590 °C of Fe–Cr alloys. Both techniques yielded the same content, approximately 6 at.%. This content proved to be an equilibrium value as further annealing for 24 h lead to coarsening of the precipitate without change in their iron concentration. This presence of iron in CrN is qualitatively consistent with the thermodynamic properties of FeN and CrN. It has implications on the phase diagram of the Fe–Cr–N ternary system, which should be reassessed in order to better understand the nitriding process and better model the precipitation kinetics of CrN. Iron in CrN, however, did not affect the Cr/N ratio in the precipitates and, thus, cannot explain excess nitrogen, either in whole or in part. It was further shown using 3D calculations that only part of this excess nitrogen can be due to misfit-induced elastic stresses and that further investigation remains necessary to provide a complete explanation.

## Figures and Tables

**Table 1:** Atomic compositions of the Fe–Si alloy, after cold rolling, nitriding, and annealing. Compositions in the annealed sample are given separately for the ferritic matrix and Si_3_N_4_ precipitates. Al–N clusters are also excluded for the calculation of matrix composition. The error is calculated from counting statistics, according to [35].

Alloying Elements	Cold Rolled	As-Nitrided	Annealed	
			Matrix	Si_3_N_4_
Fe	96.77 ± 0.01	96.82 ± 0.01	97.98 ± 0.01	0.10 ± 0.02
Si	3.10 ± 0.01	2.91 ± 0.01	1.79 ± 0.01	42.03 ± 0.35
^15^N	-	0.18 ± 0.01	0.13 ± 0.01	55.71 ± 0.35
Al	0.06 ± 0.01	0.06 ± 0.01	0.04 ± 0.01	1.75 ± 0.09
Ti	0.04 ± 0.01	0.03 ± 0.01	0.03 ± 0.01	0.27 ± 0.04
Mn	0.01 ± 0.01	0.01 ± 0.01	-	0.14 ± 0.03
C	0.02 ± 0.01	0.01 ± 0.01	0.03 ± 0.01	-

**Table 2:** Physical properties of ferrite (bcc-iron) and chromium nitride (fcc-CrN) used as input for the mechanical calculations.

Physical Properties	α-Fe	CrN
Young modulus (GPa)	178	230
Poisson coefficient	0.3	0.29
Molar volume (cm^3^/mol)	7.16	11.30
Volume weight (g/cm^3^)	7.8	5.9
